# RETRACTION: Prophylactic Sacral Protective Dressings' Effect on Preventing Pressure Injury: A Meta‐Analysis

**DOI:** 10.1111/iwj.70336

**Published:** 2025-03-17

## Abstract

**RETRACTION:** GongX. and XuR. , “Prophylactic Sacral Protective Dressings' Effect on Preventing Pressure Injury: A Meta‐Analysis,” International Wound Journal 19, no. 6 (2022): 1463–1470, 10.1111/iwj.13743.34962078 PMC9493227

The above article, published online on 27 December 2021, in Wiley Online Library (http://onlinelibrary.wiley.com/), has been retracted by agreement between the journal Editor in Chief, Professor Keith Harding; and John Wiley & Sons Ltd. Following an investigation by the publisher, all parties have concluded that this article was accepted solely on the basis of a compromised peer review process. The editors have therefore decided to retract the article. The authors did not respond to our notice regarding the retraction.

**RETRACTION:** X. Gong and R. Xu , “Prophylactic Sacral Protective Dressings' Effect on Preventing Pressure Injury: A Meta‐Analysis,” International Wound Journal 19, no. 6 (2022): 1463–1470, 10.1111/iwj.13743.34962078 PMC9493227

The above article, published online on 27 December 2021, in Wiley Online Library (http://onlinelibrary.wiley.com/), has been retracted by agreement between the journal Editor in Chief, Professor Keith Harding; and John Wiley & Sons Ltd. Following an investigation by the publisher, all parties have concluded that this article was accepted solely on the basis of a compromised peer review process. The editors have therefore decided to retract the article. The authors did not respond to our notice regarding the retraction.

